# Possible Interaction Between Hibiscus and Warfarin Resulting in Severe International Normalized Ratio Elevation: A Case Report

**DOI:** 10.7759/cureus.109805

**Published:** 2026-05-28

**Authors:** Khaled Bani Yaseen, Mohammad Al-Radaideh, Majed Almadaineh, Ahmad W Haddad

**Affiliations:** 1 Internal Medicine, Saint Michael’s Medical Center, Newark, USA

**Keywords:** anticoagulation, cytochrome p450 2c9, drug interaction, herb–drug interaction, hibiscus, mechanical valve, supratherapeutic inr, warfarin, warfarin toxicity

## Abstract

Warfarin has a narrow therapeutic window and is susceptible to interactions with medications, foods, and herbal products. We describe the case of a 59-year-old woman with a mechanical mitral valve receiving chronic warfarin therapy who developed a markedly elevated international normalized ratio (INR) after recently consuming hibiscus tea. No other clear precipitating factor for the supratherapeutic INR was identified. Warfarin was temporarily withheld, hibiscus intake was discontinued, and oral vitamin K was administered with subsequent normalization of the INR. This case highlights the importance of obtaining a thorough dietary and herbal history in patients receiving warfarin therapy and raises awareness of a possible interaction between warfarin and hibiscus products.

## Introduction

Warfarin remains one of the most commonly prescribed oral anticoagulants for patients with mechanical heart valves and other thromboembolic conditions [1]. Despite its effectiveness, maintaining a therapeutic international normalized ratio (INR), which measures the blood clotting time related to the coagulation factor VII, can be challenging because warfarin possesses a narrow therapeutic index and is highly susceptible to interactions with medications, foods, and herbal products [2]. The international normalized ratio (INR) is used as an indirect measure of warfarin’s anticoagulant effect. Patients receiving warfarin require close INR monitoring, particularly during dose adjustments, as even minor changes in warfarin dose or metabolism due to interactions with medications or dietary substances may lead to significant INR fluctuations. Elevated INR levels increase the risk of bleeding, whereas low INR levels may result in inadequate anticoagulation and thrombus formation [3]. Numerous agents have been associated with potentiation of warfarin activity and the development of supratherapeutic INR levels, including macrolide antibiotics, azole antifungals, amiodarone, and several herbal products such as Ginkgo biloba and cranberry [2]. These interactions may increase the risk of clinically significant bleeding and frequently necessitate close INR monitoring and dose adjustments [3].

The use of herbal and dietary supplements has increased substantially worldwide, often without physician awareness or adequate patient counseling regarding potential drug interactions. Hibiscus products, particularly hibiscus tea, are widely consumed because of their perceived cardiovascular and antihypertensive benefits [4]. Experimental studies have suggested that hibiscus may influence cytochrome P450 enzyme activity and drug metabolism, raising concern for possible interaction with medications metabolized through similar pathways, including warfarin [5]. However, clinical evidence describing a potential interaction between warfarin and hibiscus remains scarce, with only limited case-based observations reported in the literature [5].

We present the case of a 59-year-old woman with a mechanical mitral valve on chronic warfarin therapy who developed a markedly elevated INR following recent ingestion of hibiscus tea. The absence of other identifiable causes of INR elevation and the temporal relationship between hibiscus consumption and the supratherapeutic INR suggest a possible association. This case highlights the importance of obtaining a detailed dietary and herbal history in patients receiving warfarin therapy and underscores the need for further investigation into the safety of commonly consumed herbal products.

## Case presentation

A 59-year-old woman with a past medical history of type 2 diabetes mellitus, hypertension, hyperlipidemia, hyperthyroidism, and rheumatic heart disease status post-mechanical mitral valve replacement in 2013 presented to the emergency department after routine laboratory testing revealed a markedly supratherapeutic INR. She was asymptomatic and denied any bleeding, headache, visual disturbances, or dizziness. The patient was a lifelong non-smoker and denied alcohol consumption or illicit drug use. She had no known allergies. She lived with her daughter and reported good social support. Physical examination showed an alert, oriented patient in no acute distress. Neurological examination was unremarkable. Cardiac auscultation revealed a metallic click without murmurs. Lung examination was clear, the abdomen was soft and non-tender, and the skin showed no bruising or ecchymosis. Vital signs were stable, with a temperature of 36.9°C, a regular heart rate of 84 beats/minute, a respiratory rate of 17 breaths/minute, blood pressure of 124/78 mmHg, and oxygen saturation of 98% on room air.

Initial laboratory evaluation demonstrated normal complete blood count, renal function tests, liver function tests, B-type natriuretic peptide (BNP), high-sensitivity troponin, HbA1c, and low-density lipoprotein cholesterol levels. Thyroid studies revealed decreased thyroid-stimulating hormone and increased free T4 levels. Coagulation studies demonstrated markedly elevated INR and prolonged prothrombin time. Lab tests are shown in Table 1.

**Table 1:** Laboratory findings. INR = international normalized ratio; PT = prothrombin time; BNP = B-type natriuretic peptide (BNP); TSH = thyroid-stimulating hormone; LDL = low-density lipoprotein

Lab test	Patient’s values	Reference range
INR	11.9	0.8–1.2
PT	126.5 seconds	11–14 seconds
BNP	49 pg/mL	<100 pg/mL
High-sensitivity troponin	27 ng/L	<34 ng/dL
Free T4	3.75 ng/dL	0.8–1.8 ng/dL
TSH	<0.01 mIU/L	0.45–4.5 mIU/L
LDL	37 mg/dL	<100 mg/dL
HbA1c	5.6%	<5.7%

Non-Contrast CT of the head showed no acute intracranial abnormalities (Figure 1).

**Figure 1:**
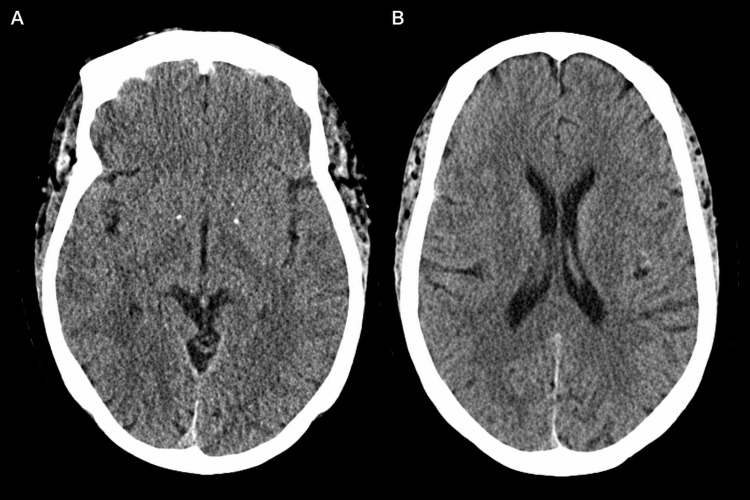
Non-contrast CT head images demonstrating no acute intracranial abnormalities, including no evidence of intracranial hemorrhage, mass effect, or midline shift.

Further history revealed chronic warfarin therapy with alternating doses of 10 mg and 7.5 mg for the past year, during which her INR had remained stable around the target of 3.5. Her medications included aspirin 81 mg daily, metoprolol tartrate 25 mg twice daily, atorvastatin 40 mg daily, and methimazole 10 mg daily. She denied recent medication changes and reported adherence to a warfarin-appropriate diet. However, she admitted to consuming a small cup of hibiscus tea two days before INR testing after hearing about its perceived health benefits.

Warfarin was held, and she received a single 5 mg dose of oral vitamin K solution. Repeat testing the following day showed improvement of INR to 5.3. She was discharged on warfarin 7.5 mg daily with close outpatient follow-up and was advised to avoid hibiscus while taking warfarin, given the suspected association with the severe INR elevation. One week later, her INR was 2.5, and her previous alternating regimen of 10 mg and 7.5 mg was resumed. Repeat INR testing one week later showed a therapeutic INR of 3.4, and she was advised to continue the same regimen.

Figure 2 shows the INR trend from admission until stabilization on the patient’s previous maintenance dose of warfarin.

**Figure 2:**
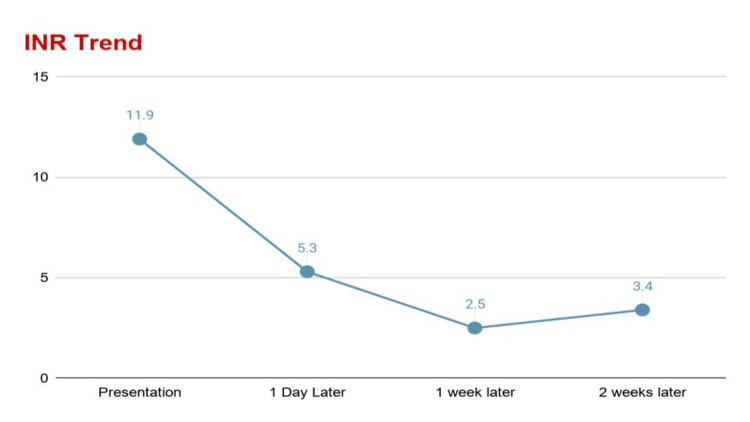
INR trend. INR = international normalized ratio

## Discussion

Patients with mechanical heart valves require lifelong anticoagulation with warfarin [1]. Despite the development of newer non-vitamin K oral anticoagulants, studies have not demonstrated superiority over warfarin in this population; in fact, some studies have reported increased risks of valve thrombosis and systemic embolization with their use in patients with mechanical valves [1,6]. One of the major challenges of warfarin therapy is the need for frequent INR monitoring [3]. In addition, warfarin is known to interact with numerous agents, including medications, dietary products, and herbal supplements [2]. Therefore, careful attention must be given to patient counseling regarding potential interactions and substances that should be avoided while on warfarin therapy.

Warfarin is primarily metabolized by the hepatic cytochrome P450 enzyme CYP2C9 [7]. The activity of this enzyme can be altered by numerous agents, including medications and herbal products. Several substances inhibit CYP2C9, thereby potentiating the anticoagulant effect of warfarin and increasing INR levels, including azole antifungals, macrolide antibiotics, amiodarone, Ginkgo biloba, and cranberry products [2]. In contrast, agents such as rifampin, carbamazepine, and St. John’s wort induce CYP2C9 activity, leading to enhanced warfarin metabolism and reduced anticoagulant effect [8-10].

In recent years, consumption of hibiscus tea has increased because of its reported beneficial effects on blood pressure and lipid profile [4]. Only a few cases in the literature have described a possible association between hibiscus ingestion and warfarin toxicity [5], making it difficult to establish a definitive causal relationship. The proposed mechanism involves inhibition of cytochrome P450 2C9 by hibiscus [5]. As warfarin is primarily metabolized through this pathway, inhibition of CYP2C9 may reduce warfarin metabolism and potentiate its anticoagulant effect [5]. Nevertheless, the clinical significance of this interaction remains uncertain, as current evidence is largely derived from in vitro studies, and human data remain limited [5].

In our patient, the absence of identifiable common causes of INR elevation, along with the temporal relationship between hibiscus ingestion and the marked INR elevation, raised concern for a possible herb-drug interaction. Furthermore, the patient’s INR had remained stable on the same warfarin regimen before hibiscus consumption and subsequently normalized and remained therapeutic after discontinuation of hibiscus without major adjustment of warfarin dosing, further supporting hibiscus as the likely contributing factor. Although hyperthyroidism can potentiate the anticoagulant effect of warfarin through increased turnover of vitamin K-dependent clotting factors [11], our patient had maintained stable INR values on the same warfarin regimen despite persistently abnormal thyroid function tests over an extended period before hibiscus ingestion, making thyroid dysfunction alone less likely to explain the abrupt and marked INR elevation.

## Conclusions

Warfarin remains the first-line and virtually the only approved oral anticoagulant for patients with mechanical heart valves. Nevertheless, its use is complicated by the need for frequent INR monitoring and its numerous drug, dietary, and herbal interactions. In this case, hibiscus was suspected to be a potential contributor to warfarin toxicity, resulting in a markedly supratherapeutic INR and increased bleeding risk. Therefore, in any patient receiving warfarin who presents with either supratherapeutic or subtherapeutic INR levels, obtaining a detailed history regarding recent dietary changes, herbal supplement use, medication intake, and treatment adherence is essential. Recognition of potential interacting factors may help prevent future INR instability and avoid unnecessary medication adjustments or alterations in anticoagulation therapy.
